# Multireference Fock Space Coupled-Cluster Method for the (3,0) Sector

**DOI:** 10.1021/acs.jpca.4c04357

**Published:** 2024-10-25

**Authors:** Monika Musial, Stanisław A. Kucharski

**Affiliations:** Institute of Chemistry, University of Silesia in Katowice, Szkolna 9, Katowice 40-006, Poland

## Abstract

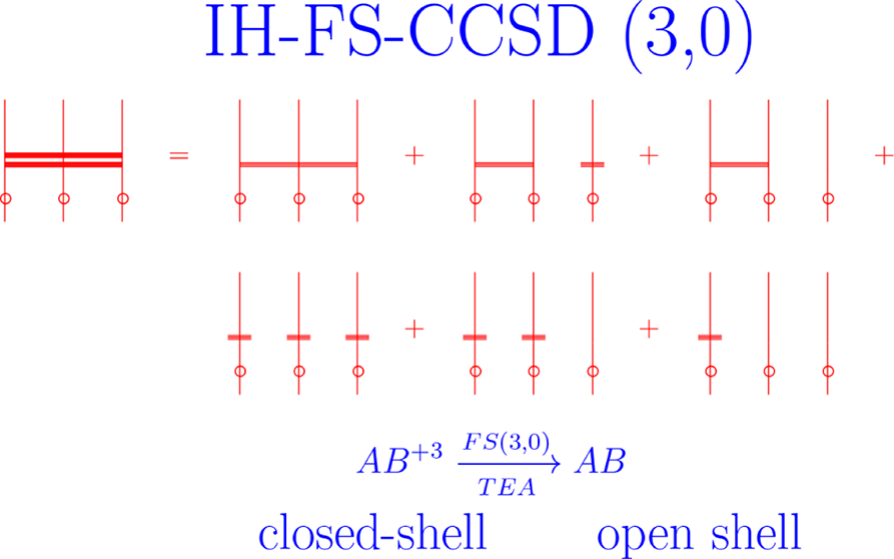

This work reports an implementation of a novel realization of the multireference coupled cluster theory formulated in Fock space. Extending the previous formulation carried out in the (1,1) [M. Musial, R. J. Bartlett, J. Chem. Phys. **129**, 044101 (2008)], (0,2) [M. Musial, R. J. Bartlett, J. Chem. Phys. **135**, 044121 (2011)], and (2,0) [M. Musial, J. Chem. Phys. **136**, 134111 (2012)] sectors to the (3,0) sector, we are able to treat structures with three valence electrons. The (3,0) sector describes systems with three electrons added to the reference, which means that in order to perform correlated calculations for the neutral *AB* molecule, we have to adopt as the reference a triply ionized structure *AB*^3+^. A desirable situation occurs when such an ion has a closed-shell structure and also dissociates into closed shell fragments. This feature makes it possible to apply the restricted Hartree–Fock scheme for the whole range of interatomic distances. Examples of molecules of this type are the diatomics formed by the atoms of alkali metals and alkaline earth metals. An analogous structure is also exhibited by alkali metal monocarbides. In the current work, we have calculated the potential energy curves and spectroscopic constants for the LiBe, LiC, and NaC molecules.

## Introduction

This study is a continuation of previous works devoted to the quantum chemical description of the bond-breaking process and focused on the accurate description of potential energy curves (PECs) and spectroscopic constants of selected diatomic molecules.^1−6^ Our purpose was to develop a first-principles method that would generate PECs for both the ground and excited states of diatomics. The correct description of a dissociation process remains a challenging issue in quantum chemistry. The main difficulty is connected with the fact that in ahomolytic dissociation of the standard (say single) bond, we encounter a closed-shell structure around the equilibrium and an open-shell one, when approaching a dissociation limit. This forces us to use the UHF (unrestricted Hartree–Fock) reference function with its well-known deficiencies such as undetermined spin or problems with converging the post-HF solutions. A remedy for this situation would be to use as the reference structure an ionic form of the original *AB* molecule selected in such a way that both the equilibrium structure and the dissociation products are described by the closed-shell function. In the case of alkali metal diatomics, this was a doubly ionized cation *AB*^2+^.

However, the critical step in such a procedure is the selection of the method, which at the correlated level recovers the original neutral structure. Here, we reach for the multireference (MR) coupled cluster (CC) method formulated in Fock space. A comprehensive presentation of the main aspects of the MR theory in relation to the single reference approach has been provided by Bartlett.^7^ The characteristic feature of the MR method is its flexibility toward the selection of the reference functions. Depending on the chosen sector of the Fock space, we have the freedom to select a reference with a variable number of valence electrons. In the case of the alkali metal diatomics aforementioned, the proper FS sector is (2,0), which means that the FS-MRCC at the correlated level generates results for the structure with two electrons added to the reference adopted at the HF level. In other words, if we adopt as the reference a doubly ionized structure *AB*^2+^, then the FS-MRCC(2,0) will provide the results pertaining to the neutral *AB* system. It was shown in several papers that this model works quite well. For example, for the Li_2_ molecule,^3^ we have presented PECs and spectroscopic constants for 34 lowest-lying electronic states. The average deviation from the experiment for the excitation energy (EE) is equal to 0.005 eV which is less than 0.3%. We have obtained lower but also a relatively high accuracy for the PECs for other singly bonded structures, i.e., NaLi,^4^ where the mean average error for the excitation energy was equal to 0.007 eV. It should also be mentioned that the theoretical potential energy curves match the experimental ones very well, reproducing with high accuracy each particular potential well, also in the states with multiple avoided crossings.

The aim of the current study is to implement a novel variant of the FS-CC method formulated in the three-valence sector, i.e., (3,0),^8−10^ capable of describing atomic or molecular species with three electrons added to the reference system. Applying the FS-CC in the (3,0) sector to the neutral system, say *AB* (we get the HF solution for *AB*), we obtain results concerning the triply negative ion *AB*^3–^. Assuming as a reference the triply positive ion *AB*^3+^ (i.e., solving HF equations for this ion), we obtain at the correlated level (i.e., using the FS-CC (3,0) scheme) the results for the neutral *AB*. So if, for example, there is a situation in which the triply ionized molecule assumes a closed-shell structure, and in addition, it dissociates into closed-shell fragments, we may use the FS-CC (3,0) method to produce its PECs on the basis of the RHF (restricted HF) function. An example is an open-shell LiC molecule with its quartet ground state (GS) which – triply ionized – dissociates as  Li^+^+C^2+^, i.e., generates closed-shell cations Li^+^ and C^2+^. Other neutral structures with the same property are diatomics of the type LiBe, LiMg, NaBe, and others, formed by alkali and alkaline earth metals. Suitable objects are formed by cations of alkaline earth metal diatomics (e.g., , ) as well as anions of the alkali metal dimers (, ).

## Methods

The studies of the PECs of diatomic molecules have a well-established place in the quantum chemical literature. The problem is challenging since, as was mentioned in the previous section, upon a dissociation of the closed-shell structure, usually the open-shell fragments are formed, complicating the calculations at the correlated level. This is why, for example, in the case of the dissociation of the alkali metal diatomics, the dominant approach is based on the effective core potential (ECP), which replaces electrons of the inner shells with model potentials and owing to that reduces the correlated calculations to two valence electrons. Our concept – based on the first-principles approach – relies on adopting the ionized (positively) structure as a reference system, selected in such a way that its dissociation products are closed-shell fragments. The next step – within this strategy – requires a proper version of the FS-CC approach capable of recovering the original neutral structure. We give some rudiments of the method we implemented and used in the following.

### Basic Definitions of The Multireference CC Formalism

Within the single reference (SR) theory, the wave function (eq 1) is defined via an exponential ansatz^11−28^
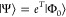
1where *T* is the cluster operator defined as in eq 2:
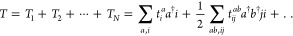
2Operators *T*_1_,···,*T*_*N*_ are responsible for single,···, *N*-tuple excitations from the reference ; the  are the second-quantized operators removing electron from the occupied level *i* and placing it on the virtual one *a*. The  amplitudes are the solution of the CC equations obtained by a projection of the  operator against excited configurations :
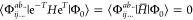
3In our case, i.e., at the CCSD level,  and  is represented by  and .

The multireference^29−47^ formalism assumes that the configurational space is divided into two subspaces: the model space with the projector operator *P* and its orthogonal complement defined by the projector *Q*. The advantage of the MR approach lies in the fact that the diagonalization of the Hamiltonian *H* within the full configurational space (size: millions, billions, ...) is replaced by the diagonalization of the effective Hamiltonian *H*_eff_ operator (eq 4) in the model space. The size of the model space is drastically smaller, and the diagonalization of the *H*_eff_ can be carried out in most cases with standard diagonalization techniques.

The effective Hamiltonian is defined as
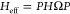
4where Ω is a wave operator, which when acting on the model function  generates an exact wave function (eq 5):
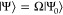
5In order to use particle–hole second-quantized operators, the configuration adopted as a Fermi vacuum must be selected. Then, the model determinants can be generated by the action of the particle–hole creation operators within the active space formed by the valence one-particle levels. The important parameters defining the active space are its size denoted as *K* and the number of valence electrons denoted as *N*_v_.

The MR-CC considered here, i.e., formulated in the Fock space (also known as valence universal)^39,46^, admits the configuration with a variable number of valence electrons. The FS model is obtained by a distribution of 0, 1, 2, ..., *N*_v_ valence electrons among *K* valence orbitals. It is obvious that it includes configurations containing 0 valence electrons i.e., with *N*_*v*_ electrons removed from the system as well as *K* valence electrons, i.e., -tuply ionized anion. The valence universality means that (i) all model configurations are defined with respect to the same Fermi vacuum and (ii) the wave operator Ω is defined identically for all reference determinants.

An important step in the FS approach relates to the selection of the Fermi vacuum which – on one hand – defines the reference system for which the one-particle states are obtained (in our approach, the Hartree–Fock solutions) and–on the other–determines the sector structure of the model space. The sector is defined by the number of valence particles and valence holes created with respect to the vacuum, e.g., a (*k*, *l*) sector indicates that the reference configuration contains *k* valence particles and *l* valence holes.

The flexibility of the FS approach – as indicated already in the previous section – can be used in all situations where we want to replace the open-shell reference (requiring the UHF function) with the closed-shell one, conveniently described by the RHF method. The simplest example would be the dissociation of, e.g., the Li_2_^+^ ion. We can imagine three different ways (remaining within the CC theory) to obtain the desired curves: (i) using the single reference CC scheme based on the UHF solution obtained for Li_2_^+^, (ii) using the FS-CC (0,1) based on the HF solution for the Li_2_ dimer: we could use the RHF solution around the equilibrium and switch to the UHF for larger distances, and (iii) using the FS-CC (1,0) variant based on the RHF solution for all interatomic distances of the Li_2_^2+^ ion (dissociating into the closed-shell fragments).

In the current work, we develop an approach based on the three-valence sector, denoted as (3,0), which is obtained by adding three valence electrons to the Fermi vacuum, i.e., the zero-valence sector indicated as (0,0).

### General Characteristics of the Fock-Space Coupled-Cluster Approach

As shown above, the Fock space approach provides the desired flexibility in selecting a reference system which – on one hand – is of closed-shell character at equilibrium and – on the other hand – dissociates into closed-shell fragments at the asymptotic limits.

The basic general formula for the MR approach is a Bloch equation of the form
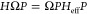
6Operating from the left with the *P* operator, we obtain the expression for the *H*_eff_ () (the relation  has been used), while the projection against the configurations belonging to the orthogonal subspace (represented by the *Q* operator) generates MR-CC equations. The Ω operator can be expressed as in eq 7:
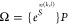
7where the braces  indicate that the normal ordering is imposed on the product of  operators, and  includes all operators of lower sectors (eq 8):
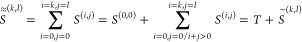
8The  is the excitation operator which can be written as a sum of operators (eq 9):
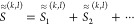
9and, in general,  can be defined as follows (eq 10):
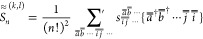
10where ′ indicates that the excitations within a model space are excluded. The *n* index in the last expression indicates the number of creation (annihilation) operators. We introduce the index notation with the following relations between subsets of indices eq 11 and eq 12:
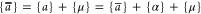
11
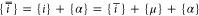
12Indices  () run over active and inactive particles (holes), i.e., levels unoccupied (occupied) in ;  () run over inactive particles (holes);  () run over active particles (holes);  () run over all active levels and inactive particles (holes).

The FS-CC equations, obtained from eq 6 by projection from the right onto the reference determinant and from the left onto the orthogonal space configuration, can be written as
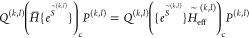
13where replacing the  with , i.e., factoring out e^*T*^, made it possible to replace the *H* operator with its similarity transformed counterpart: . The model space projection produces the effective Hamiltonian (eq 14):
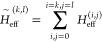
14and the subscript c means connected terms only.

### Fock Space Equation for the (3,0) Sector

It needs to be mentioned that there is a fundamental difference between the single-reference CC equations, as given in eq 3, and the FS-MR equations, as given by eq 13. In the latter case, the amplitudes from higher sectors do not contribute to the equations set up for lower ones. In the SR (single-reference) situation in the CCSD model, we solve the equations for the *T*_1_ and *T*_2_ operators, obtaining a set of CC amplitudes. When going to higher models, say, CCSDT, we again solve the equations for *T*_1_, *T*_2_, and *T*_3_. This is so since the CC equations for single reference are coupled, and inclusion into the cluster expansion of the *T*_3_ operator changes the equations for the lower rank analogues.

The FS equations are partially decoupled, i.e., the cluster operators defined for higher sectors never enter the equations constructed for the lower ones. However, it does not work the other way. This means that the *S* operators found for the lower sectors enter the equations for higher ones. Hence, e.g., in order to set up equations for the *S*^(1,1)^ amplitudes, i.e., for the (1,1) sector, we need to have access to the amplitudes from the (1,0) and (0,1) sectors. It follows from the above that the amplitudes from the (1,0) and (2,0) sectors enter the FS equations for the *S*^(3,0)^ amplitudes as constant terms determined beyond the (3,0) formalism. Hence, we may omit the presentation of the FS equations for the (1,0) and (2,0) sectors since they are given elsewhere,^1,42^ and here we focus only on the (3,0) amplitudes and corresponding elements of the effective Hamiltonian.

For the (3,0), sector the  operator at the CCSD level is defined in the following way (eq 15):
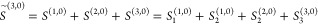
15and the FS equation for the (3,0) sector takes a form:
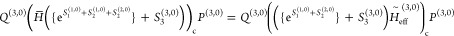
16where  is defined in eq 17:
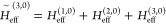
17On the right-hand-side of eq 16, we have eliminated the  component from the exponent as it does not contribute to this equation. The expressions for the  and  were already presented previously;^1,42^ hence, here we give the three-body component only:
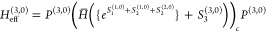
18The equations for the  amplitudes in a more expanded form are as follows:
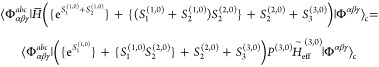
19where the indices  run over valence particle levels, and  run over the full set of particle levels defined with respect to the current vacuum (with the restriction that at least one of the indices must be inactive).

The computational procedure for solving the  amplitude equations is as follows. In the first step, we provide both *S* amplitudes and *H*_eff_ elements by solving the respective FS equations in lower sectors, described elsewhere.^1^ The solutions for the (1,0) and (2,0) sectors are transferred to the  part of the program in an unchanged form; i.e., the  and  amplitudes are treated here as known data. Next, we solve eq 19 iteratively constructing in each iteration the  operator, eq 18, to be used subsequently in eq 19. The diagonalization of the  in each iteration provides the sought energy values. It should be mentioned that the effective Hamiltonian at the (3,0) level is correct through the second order of Moeller–Plesset perturbation theory. This feature requires the correctness of *H*_eff_ also in the lower sectors. In our approach both  and  are correct through the second order. This limitation is a consequence of the restriction of the CC model to singles and doubles. This computational scheme described above is inefficient, and the FS equations are very frequently divergent.

**Figure 1:**
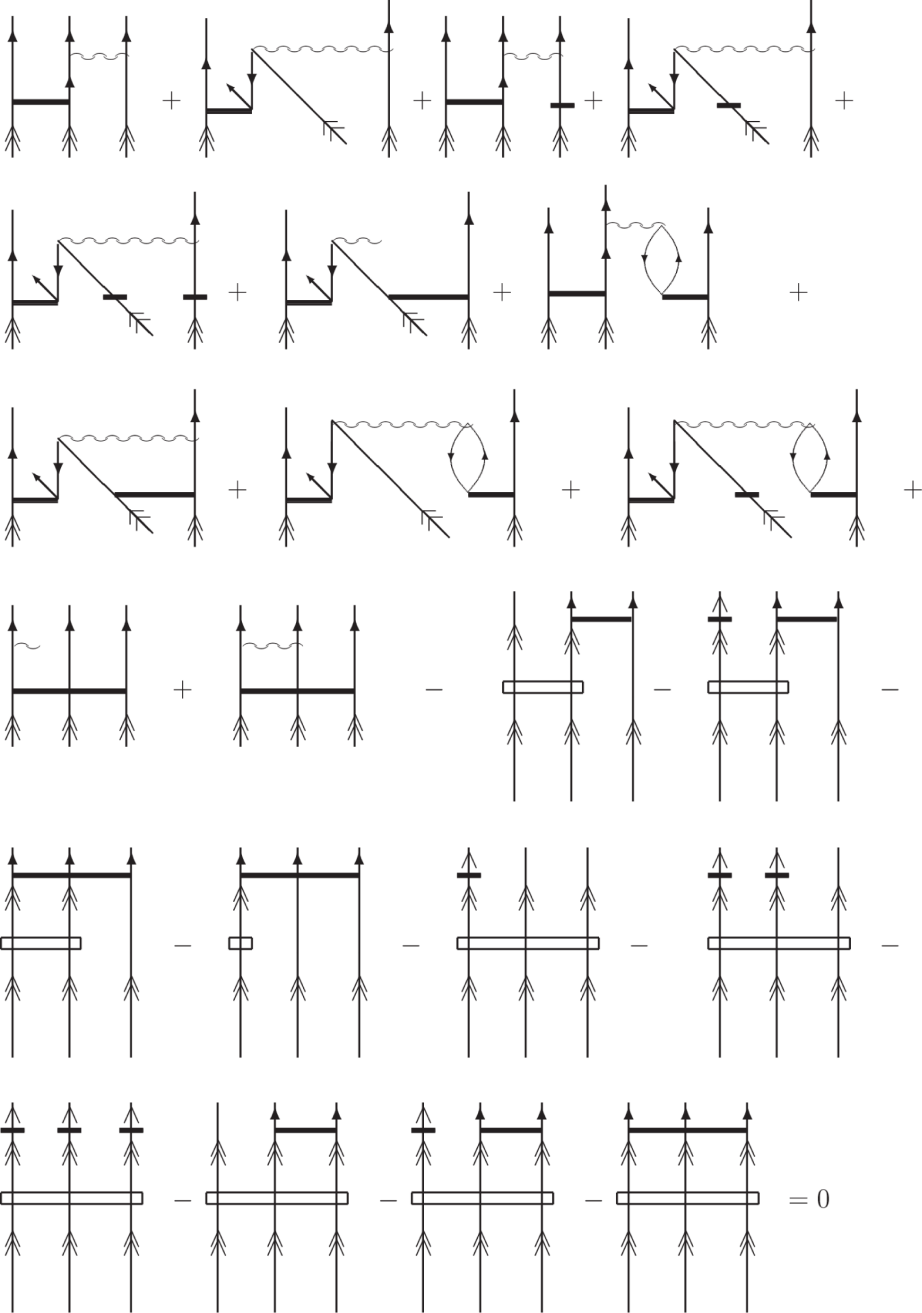
Diagrammatic representation (in antisymmetrized form) of the *S*_3_ equation for the (3,0) sector at the FS-CCSD level. The wiggly lines refer to the  elements (defined in refs (1) and (42)) and the solid horizontal lines represent the *S* amplitudes defined in Figure 2, whereas lines with rectangles represent the effective Hamiltonian elements (see Figure 2). Double arrows refer to the active lines, single full arrows indicate summation over inactive and active hole or particle labels, and single plain arrows refer to the inactive lines. The equations for the one-valence and two-valence amplitudes – as well as – the expressions for the effective Hamiltonian elements used in the  equation are defined in refs (1) and (42). An expression for  is presented in Figure 3.

**Figure 2:**
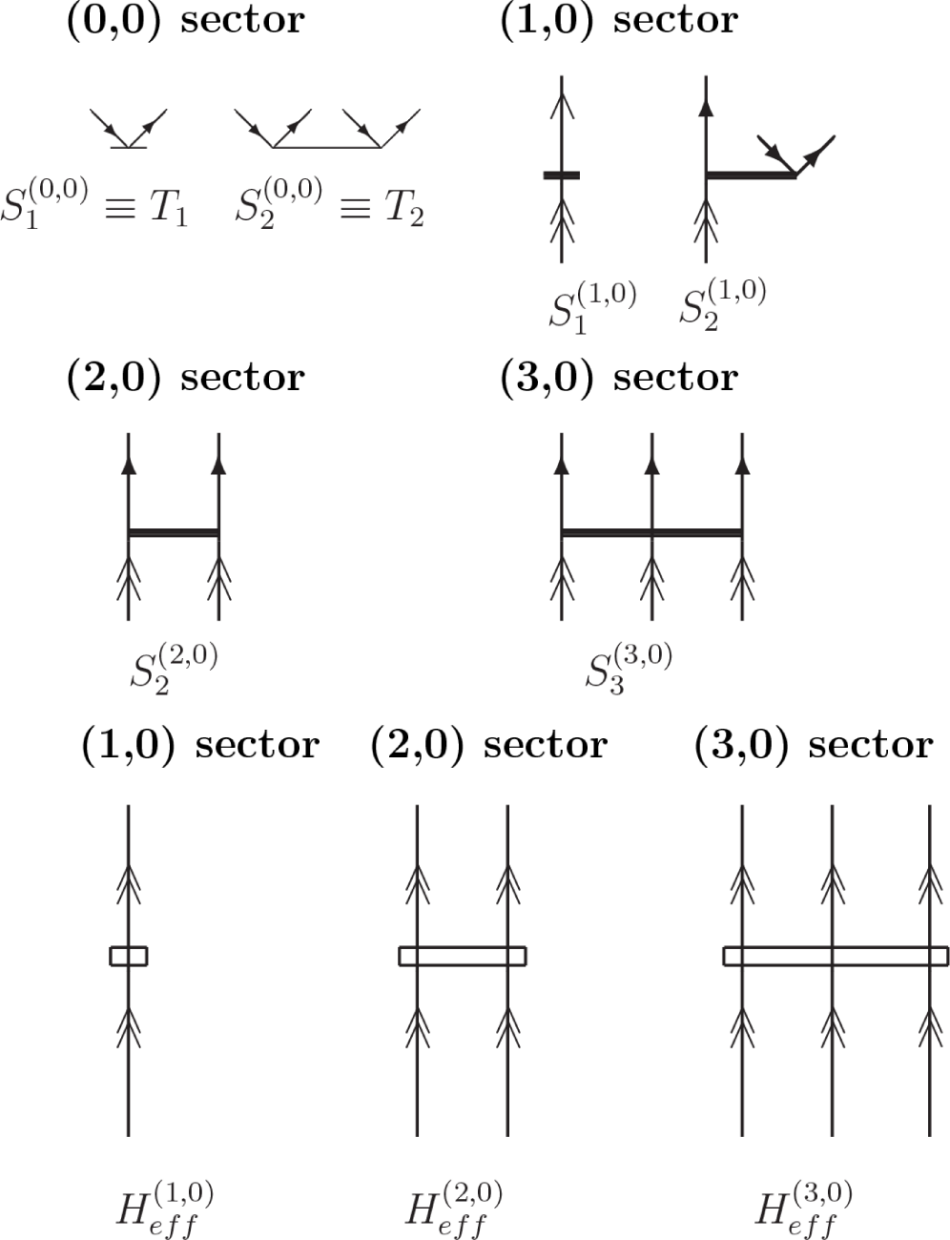
Graphical representation of the *S* amplitudes and effective Hamiltonian elements at the CCSD level. Double arrows refer to the active lines; single plain arrows refer to the inactive lines; single full arrows refer to the inactive and active lines.

**Figure 3:**
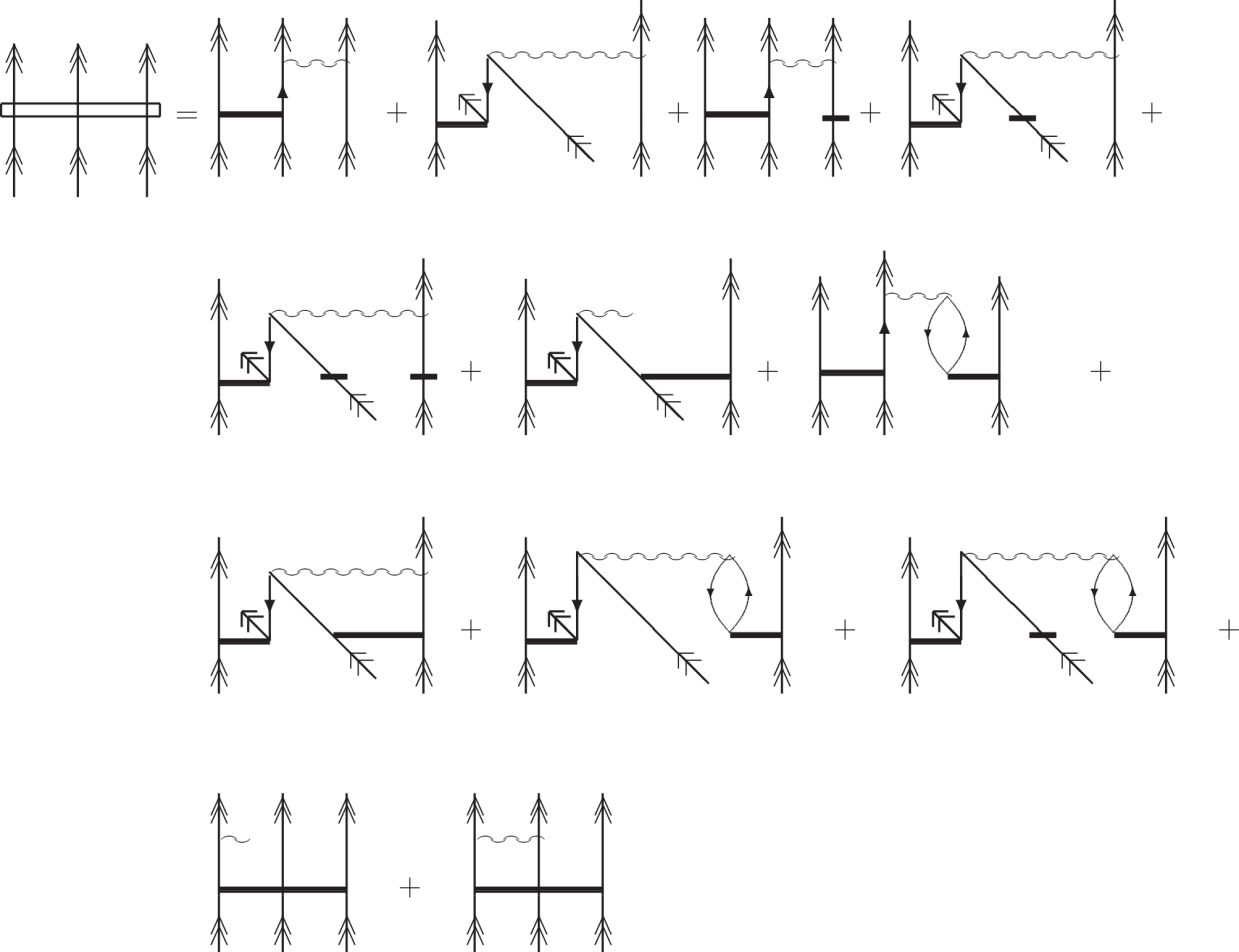
Diagrammatic representation (in antisymmetrized formalism) of  at the CCSD level (corresponding to eq 18). The wiggly lines refer to the  elements (defined in refs (1) and (42)) and the solid horizontal lines represent the *S* amplitudes.

The working equation for FS  at the CCSD level, corresponding to eq 19, in diagrammatic form (in the antisymmetrized formalism) is presented in Figure 1, while the *S* amplitudes and the effective Hamiltonian elements occurring in Figure 1 are defined in Figure 2 and 3.

In practical realization, we use a different procedure to solve the FS equations, as discussed below.

### Practical Computational Considerations

#### Intermediate Hamiltonian (IH)-Based Fock Space Approach

An important advantage of the Fock space formulation compared to other realizations of the MRCC theory based, e.g., on the Hose-Kaldor approach,^48^ is connected with the efficiency of solving the MRCC equations. An obvious way to solve the latter for both approaches is an iterative scheme, which very often fails to converge. Within the FS theory, the standard iterative solving can be replaced in a straightforward manner with a robust intermediate Hamiltonian alternative.

This is a reformulation of the FS equations made by Meissner and Bartlett in several papers,^49,50^ relying on the introduction of the so-called intermediate Hamiltonian operator. It has been demonstrated^49−51^ that the diagonalization of the matrix corresponding to the IH operator also provides the eigenvalues corresponding to the effective Hamiltonian *H*_eff_. The general idea behind the introduction of the IH approach can be explained in our case, i.e., the (3,0) sector, as follows. We consider a modified  operator, denoted as ,constructed in the following way:
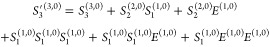
20where the *E*^(1,0)^ represents a so-called spectator line defined as in eq 21:
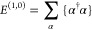
21An introduction of the latter form of the  (eq 20) operator into the Bloch equation will result in the amplitude equation being linear with respect to the . In this way, we obtain the FS equations reduced to the Bloch equations used to diagonalize the matrix via the so-called partition technique. The matrix diagonalized in that way is just the IH matrix. Although the IH matrix is significantly larger in size ( – 1)/2, *n*_v_ is the number of virtual levels) than the *H*_eff_ matrix ( – 1)/2, *K* is the number of active levels); employing the IH variants is beneficial in the context of the successful solution of the FS equation. In the current realization, we used both IH and *H*_eff_ approaches (they provide identically the same solution); however, the latter could be used successfully only for atomic calculations with small basis sets, and we have used this variant mainly for the debugging purposes.

#### Equation-Of-Motion vs Multireference Formalism

The other variant of the CC method which can be applied in the studies of excited, ionized, and electron attached states is the equation-of-motion (EOM) approach.^52−65^ A crucial quantity in both variants is a similarity-transformed Hamiltonian . The construction of the  operator requires three steps, the same for EOM-CC and FS-CC. In the first step, we obtain the reference function (by applying the Hartree–Fock method); next, we solve the single-reference CC equations to get the *T* amplitudes and the last step is a construction – on the basis of the found *T* amplitudes – of the similarity-transformed Hamiltonian .

A close computational relationship between FS-CC and EOM-CC is more clearly revealed when we consider an intermediate Hamiltonian formulation of the FS-CC scheme. The FS-CC(1,0) sector corresponds to the EA (electron affinity) realization of the EOM approach. The EA-EOM matrix diagonalized in the EOM calculation is an IH matrix in the FS-CC(1,0) formulation. Hence, the results of FS-CC(1,0) are the same as those obtained with the EA-EOM scheme.

For the (2,0) sector of the FS-CC scheme some correspondence can be found with the DEA-EOM (double electron affinity – EOM) approach.^53^ In this case, the IH (2,0) matrix is obtained from the DEA-EOM matrix by modifying part of its elements with a so-called dressing. Hence, the DEA-EOM results and FS-CC (2,0) results are different. However, in both cases, we diagonalize the matrix of the same size ( at the CCSD level).

A similar situation occurs for the (3,0) sector of the FS-CC and TEA-EOM (triple electron affinity – EOM)^59^ method. To the elements of the TEA-EOM matrix of size  – 1)/2, we have to add some corrections (dressing) to obtain the IH matrix of the FS-CC (3,0) approach.

#### IH Matrix Diagonalization

The realization of the FS method via intermediate Hamiltonian for the lower sectors (i.e., one- and two-valence) has a well-established diagonalization scheme. For the (1,0) sector, we use the Davidson scheme^66^ (modified for non-Hermitian matrices by Nakatsuji et al.^67^ ), while the (0,1) can be treated with the general QR algorithm, providing a full spectrum for a non-Hermitian matrix (available from the LAPACK library). For the two-valence sector, the QR technique is fully applicable to the (0,2) sector^2^ and, under certain conditions, also for the (2,0) sector.^1^ Generally, one may say that if the number of virtuals is on the order of 100 or less, the C_1_ molecules can be treated with the QR method. If the molecule has higher symmetry and up to 400 virtual levels, the QR diagonalization is still applicable, in particular if singlet and triplet states are diagonalized separately.

The situation in the (3,0) sector is slightly more complex. First, the QR algorithm is applicable only to small basis sets since the size of the IH matrix grows as (*n*_v_ – 1)/2 (with possible reduction when doublet-quartet separation is introduced); second, the standard Davidson procedure generally works well; however, its efficiency often depends on the quality of the starting vector. To alleviate these difficulties, we have introduced two modifications. The first one relies on reworking that part of the QR code, which generates the Hessenberg matrix. This resulted in significantly speeding up of the construction of the Hessenberg tridiagonal. The second modification is aimed at the stabilization of the Davidson diagonalization procedure. The current version that is successfully used in the (3,0) calculations is a combination of the QR algorithms and the Davidson scheme. The regular input data include the size of the active space plus all the data defining the atom or molecule and, finally, the level of calculations. In the modified variant, we also added a parameter indicating the size of some auxiliary space. The latter is a subspace of the IH space small enough to be treated with the QR algorithm, introduced just in order to provide a reliable starting vector for the Davidson iterative procedure. For example, if the size of the IH matrix is on the order of 100 000, then the auxiliary matrix of the order up to 10 000 serves this purpose well, and we may expect stable convergence of the Davidson iterations.

## Results and Discussion

The IH-FS-CCSD (3,0)/(2,0) calculations were performed using the ACES II^68^ software package augmented with our own local modules.^1,4,69^ developed to include the (3,0) sector. In this work, we present some initial applications of the implemented method, i.e., calculations of atomic term energies for the nitrogen and phosphorus atoms, calculations of equilibrium geometries (*R*_e_), harmonic frequencies (ω_e_), and adiabatic excitation energies (*T*_e_) for the LiBe, LiC, and NaC molecules; additionally, as a byproduct of the IH-FS-CCSD (3,0) calculations, we get values of *R*_e_, ω_e_, and *T*_e_ for the LiBe^+^ molecular cation. In all calculations, we used the correlation consistent pCVQZ basis set for atomic calculations and the pCVTZ basis set^70^ for molecular ones in the spherical representation. All electrons were correlated, and all the results discussed in this section are obtained at the CCSD level based on the RHF reference for the *A*^+3^ or *AB*^3+^ system. In the case of LiBe, for comparison purposes, we also performed calculations using standard UHF CC methods for the ground state, such as CCSD,^14^ CCSD(T),^18^ and CCSDT.^19^

The results, i.e., excitation energies for two doublet states, obtained for the N and P atoms are collected in Tables 1 and 2, respectively. For both atoms we compare our results with the experimental data.^71,72^ The agreement with the experiment for the nitrogen atom is very good. The ^2^*D* and^2^*P* terms are off by 0.01 eV. For thephosphorus atom the ^2^*D* term is off by 0.01 eV and^2^*P* by 0.80 eV.

**Table 1:** Excitation Energies (eV) for the N Atom Obtained Using the IH-FS-CCSD (3,0)/cc-pCVQZ Method (Number of Active Orbitals: 4)

Sym.	IH-FS-CCSD (3,0)	Exp.
^2^*D*	2.37	2.38^a^
^2^*P*	3.57	3.58^a^

a ref (71).

**Table 2:** Excitation Energies (eV) for the P Atom Obtained Using the IH-FS-CCSD (3,0)/cc-pCVQZ Method (Number of Active Orbitals: 3)

Sym.	IH-FS-CCSD (3,0)	Exp.
^2^*D*	1.40	1.41^a^
^2^*P*	2.52	3.32^a^

a ref (72).

**Figure 4:**
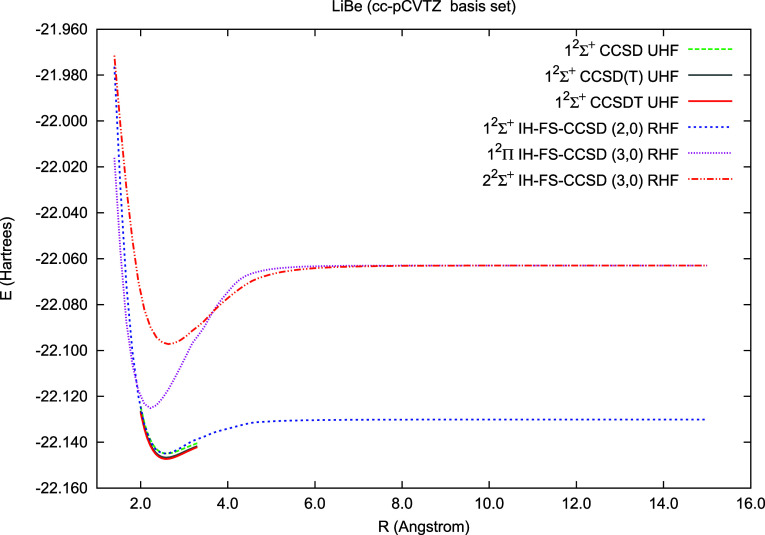
Potential energy curves of LiBe calculated using the standard CC methods (CCSD, CCSD(T), CCSDT/cc-pCVTZ) and the IH-FS-CCSD(3,0)/cc-pCVTZ method for the ground state () and two excited states:  and .

In Figure 4, we present potential energy curves for the ground () and two excited states ( and ) ofthe LiBe molecule as a function of the internuclear distance. By removing three valence electrons from the LiBe molecule, we obtain a closed-shell configuration corresponding to the LiBe^+3^ ion. Using the FS-CCSD (3,0) scheme, we recover at the correlated level the energy of the neutral LiBe molecule. In Figure 4, we plot the potential energy curves with the FS scheme and, for the ground state, also those obtained with several variants of the standard CC methods: CCSD UHF, CCSD(T) UHF, and CCSDT UHF. The most important observation is that all IH-FS-CCSD (3,0) curves go smoothly to the asymptotes, while the single reference schemes fail to converge beyond 3.5 Å. The *R*_e_, ω_e_, and *T*_e_ values for this molecule are collected in Table 3. For the ground state, they (*R*_e_, ω_e_) can be compared with the experimental values^75,76^ and other theoretical data, i.e., those given by the multireference configuration interaction method (MRCI)^73^ with 1s electrons frozen and with recent results obtained by Tomza et al.^74^ on the basis of the CCSD(T) values obtained with the 5ζ-quality basis set corrected by the estimated contributions due to full triples. The current results remain close to the experimental values (within 0.02 Å for *R*_e_ and 6 cm^–1^ for the ω_e_) and with a distance comparable to that of other theoretical values. The excited states were considered in two papers: the *R*_e_ and ω_e_ values were computed with the MRCI method in^73^ while the excitation energies *T*_e_ are obtained by the effective core potential-based calculation with the spin–orbit relativistic CI correction^77^ (denoted as SOCI) in Table 3. The current results compare very well with other theoretical values; the largest difference (ca. 90 cm^–1^) is observed for the ω_e_ value of the  state. We recalculated the latter value with the UHF-based EE-EOM-CCSD scheme and obtained the value of 538.5 cm^–1^, placed between the current and literature values.

**Table 3:** IH-FS-CCSD (3,0)/cc-pCVTZ Results for the , and  States of the LiBe Molecule (Number of Active Orbitals: 4)

Sym.	*R*_e_ (Å)	ω_e_ (cm^–1^)	*T*_e_ (eV)	Method
	2.564	319.7	-	IH-FS-CCSD (3,0)
	2.579	303.7	-	MRCI^a^
	2.572	311.8	-	CC^b^
	2.59	326.2	-	Exp.^c^
	2.226	594.0	0.542	IH-FS-CCSD (3,0)
	2.237^a^	507.5^a^	0.591^d^	MRCI^a^, SOCI^d^
	2.649	350.1	1.301	IH-FS-CCSD (3,0)
	2.594^a^	346.5^a^	1.208^d^	MRCI^a^, SOCI^d^

a ref (73).

b ref (74).

c refs (75) and (76).

d ref (77).

**Figure 5:**
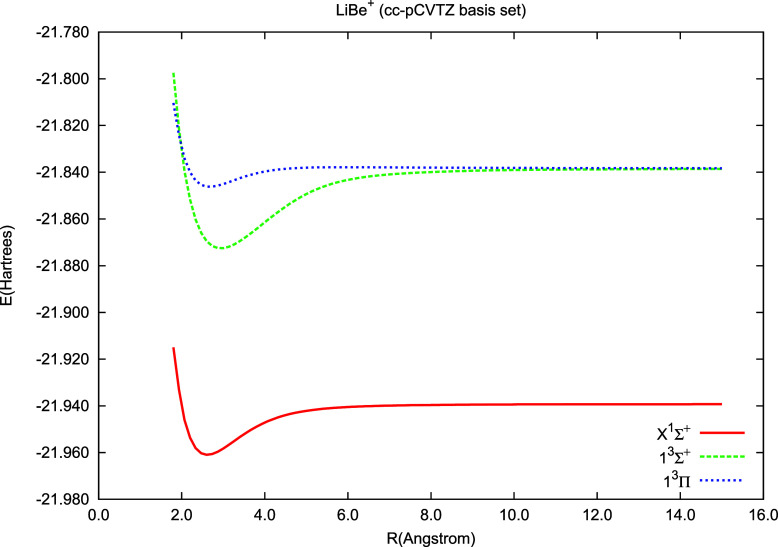
Potential energy curves of LiBe^+^ calculated using the IH-FS-CCSD(2,0)/cc-pCVTZ method for the ground state () and two excited states:  and .

As mentioned, due to the sector structure, having results for LiBe ((3,0) sector) as a byproduct, we obtain results for LiBe^+^ ((2,0) sector); we collected these results (*R*_e_, ω_e_, and *T*_e_ for the , , and  states) in Table 4 and presented PECs in Figure 5. We compare our results with experimental data^76^ and with recent theoretical ones.^78^ In ref^78^, the inner shell electrons were replaced by an effective core potential while the CISD method was used to treat two valence electrons.

**Table 4:** IH-FS-CCSD (2,0)/cc-pCVTZ Results for the , and  States of the LiBe^+^ Molecular Ion (Number of Active Orbitals: 4)

Sym.	*R*_e_ (Å)	ω_e_ (cm^–1^)	*T*_e_ (eV)	Method
	2.618	326.7	-	IH-FS-CCSD (2,0)
	2.614	323.7	-	Pseudopot.^a^
	-	313.3	-	Exp.^b^
	2.672	232.5	3.121	IH-FS-CCSD (2,0)
	2.656	244.3	3.176	Pseudopot.^a^
	2.941	289.5	2.397	IH-FS-CCSD (2,0)
	2.926	275.5	2.436	Pseudopot.^a^

a ref (78).

b ref (76).

**Figure 6:**
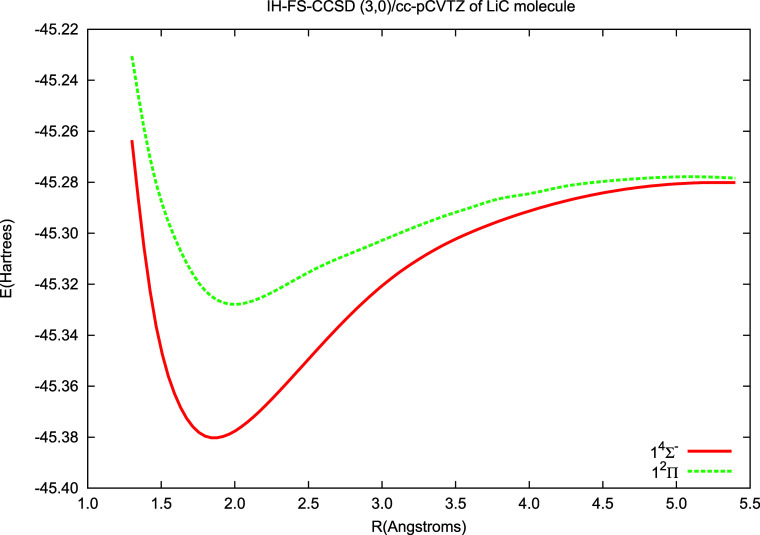
Potential energy curves of LiC calculated using the IH-FS-CCSD(3,0)/cc-pCVTZ method for the ground () and excited state ().

**Figure 7:**
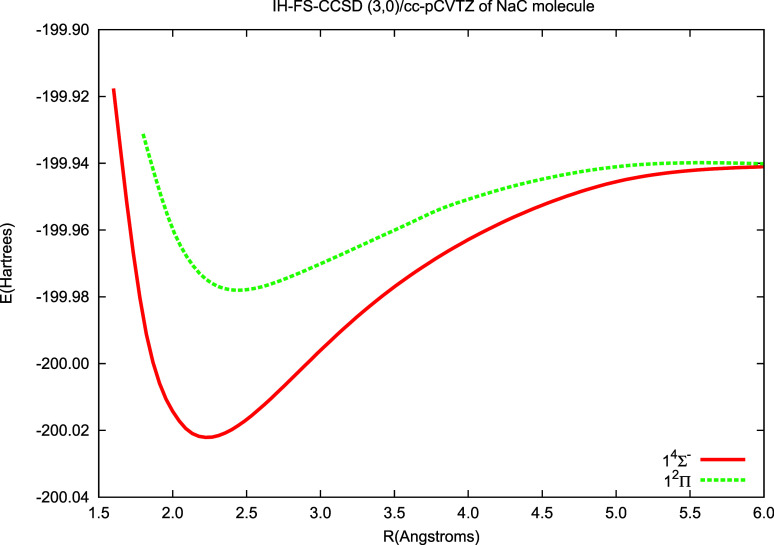
Potential energy curves of NaC calculated using the IH-FS-CCSD(3,0)/cc-pCVTZ method for the ground () and excited () state.

The results for monocarbides obtained in the current work are presented in Figures 6 and 7 and in Tables 5 and 6. The figures demonstrate the potential energy curves for the ground () state and the  excited state: Figure 6 for the LiC molecule and Figure 7 for the NaC one. In both in the ground and excited states, we observe the well-shaped minima. The experimental value is available only for the ground state of the NaC radical, which is the equilibrium bond length equal to 2.232 Å, differing from the value obtained in the current work by 0.005 Å. The other theoretical data, reported in ref^80^ and obtained with quadruple-corrected MRCI gives a slightly worse *R*_e_ value. For the excited state, the *R*_e_ and ω_e_ values are very close for both works (i.e., the current one and ref^80^) with more a pronounced difference in the case of excitation energy (0.38 eV).

**Table 5:** IH-FS-CCSD (3,0)/cc-pCVTZ Results for the  and  States of LiC (Number of Active Orbitals: 4)

Sym.	*R*_e_ (Å)	ω_e_ (cm^–1^)	*T*_e_ (eV)	Method
	1.864	685	-	IH-FS-CCSD (3,0)
	1.884	691	-	MRCI+CPP^a^
	2.004	600	1.425	IH-FS-CCSD (3,0)
	2.046	550	1.33	MRCI+CPP^a^

a ref (79).

**Table 6:** IH-FS-CCSD (3,0)/cc-pCVTZ Results for the  and  States of NaC (Number of Active Orbitals: 4)

Sym.	*R*_e_ (Å)	ω_e_ (cm^–1^)	T_e_ (eV)	Method
	2.237	475	-	IH-FS-CCSD (3,0)
	2.259	431	-	MRCI+Q^a^
	2.232	-	-	Exp.^b^
	2.445	342	1.199	IH-FS-CCSD (3,0)
	2.447	329	1.58	MRCI+Q^a^

a ref (80).

b ref (81).

For the LiC molecule, we can compare the current results with those given in ref^79^, obtained with MRCI combined with CPP (core-polarization potential). For the ground state, the differences obtained with the two methods are small (0.02 Å for *R*_e_ and 6 cm^–1^ for the ω_e_). For the  state, the equilibrium bond length differs by 0.04 Å and ω_e_ by 50 cm^–1^, with the difference in *T*_e_ staying below 0.1 eV. Similarly, as in the LiBe case, we have tried to obtain the same results with the single reference scheme using the UHF-based CCSD(T) method. For the harmonic frequency of the  state of the LiC molecule, we obtain 588 cm^–1^ and for adiabatic excitation energy 1.426 eV. Analogous values for the NaC radical are equal to 318 cm^–1^ and 1.245 eV. In both cases the single reference values remain in acceptable deviation from both the current and the literature results.

The results reported in this work are of a preliminary character. The developed computer program is not yet fully optimized; hence, both the quality of the used basis sets and the size of the active space were not optimal. We believe that further improvement of our computer code will make it possible to get more satisfying results

## Conclusions

In this work, we present for the first time the results of the Fock-space CC calculations realized fully within the (3,0) sector. This formulation opens the way to treat atomic and molecular radicals with three valence electrons. The method is particularly useful when the triple cation of the studied system has a closed-shell structure and, in addition, dissociates into closed-shell fragments. Owing to this, we can use the RHF method across the whole range of interatomic distances, which is convenient for application both at the SCF level and in correlated calculations. We implemented both the standard iterative variant based on the construction of the effective Hamiltonian (*H*_eff_) and the formulation based on the intermediate Hamiltonian (IH). In real calculations, the IH version was used. The advantage of the latter formulation is that it offers an intruder-free solution. However, the bottleneck of the IH variants is a diagonalization stage, which scales as *n*^3^ with *n* equal to (*n*_v_ – 1)/2 and *n*_v_ being the number of virtual levels.

The alkali metal monocarbides as well as the diatomics engaging atoms of the first and second groups of the periodic table are difficult systems, and the available data, both theoretical and experimental, are scarce. The calculations performed in this work provide potential energy curves and the selected spectroscopic constants for the ground and first excited states of the LiC and NaC radicals. They are, in most cases, in satisfactory consistency with other values available in the literature. A similar observation can be made for the results concerning the LiBe diatomic, for which two excited states  and  were studied. We may conclude by stating that the current work brings a first-principles tool capable of treating nonstandard chemical compounds. It can be expected that the improvement of the basis set and the enlargement of the active spaces would improve the quality of the results.
